# Diagnosing Paroxysmal Atrial Fibrillation: Are Biomarkers the Solution to This Elusive Arrhythmia?

**DOI:** 10.1155/2015/910267

**Published:** 2015-07-01

**Authors:** P. J. Howlett, F. S. Hatch, V. Alexeenko, R. I. Jabr, E. W. Leatham, C. H. Fry

**Affiliations:** ^1^School of Biosciences and Medicine, The University of Surrey, Guildford GU2 7XH, UK; ^2^Department of Cardiology, The Royal Surrey County Hospital, Guildford GU2 7XX, UK; ^3^School of Physiology and Pharmacology, The University of Bristol, Bristol BS8 1TD, UK

## Abstract

Atrial fibrillation (AF) is the commonest sustained arrhythmia globally and results in significantly increased morbidity and mortality including a fivefold risk of stroke. Paroxysmal atrial fibrillation (PAF) constitutes approximately half of all AF cases and is thought to represent an early stage of the disease. This intermittent form of atrial arrhythmia can be a challenge to identify and as a result many affected individuals are not prescribed appropriate antithrombotic therapy and hence are at risk of stroke and thromboembolism. Despite these adverse outcomes there have been relatively few diagnostic advances in the field since the introduction of the Holter monitor in 1949. This review aims to establish the available evidence for electrophysiological, molecular, and morphological biomarkers to improve the detection of PAF with reference to the underlying mechanisms for the condition.

## 1. Introduction

Atrial fibrillation (AF) is the most frequently encountered cardiac arrhythmia globally, affecting 2% of the general population and rising to 10% of those aged over 80 years. One in four individuals will experience AF in their lifetime [1]. By 2050 the prevalence of AF is expected to increase by threefold and this poses a considerable public health concern [2]. Overall AF exacerbates the risk of stroke and is associated with a twofold excess mortality with 20% of all strokes being as a result of AF and 1 in 5 patients first presenting with AF in the context of cerebral ischaemia [3]. In addition AF is also linked to larger strokes based on both clinical and radiological findings [4]. Despite these risks, it has been estimated that at least 20% of cases of AF remain undiagnosed and are not prescribed appropriate stroke prevention therapy [5].

**Figure 1:**
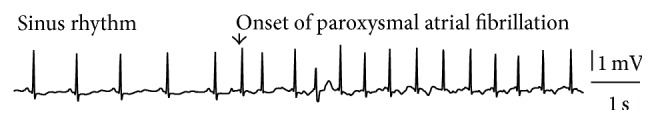
An ECG recording from a continuous cardiac monitor from a patient suffering from paroxysmal atrial fibrillation (PAF) lasting for several hours. The arrow marks the onset of AF, characterised by a variable R-R interval (representing the time between two successive ventricular contractions) and loss of P-waves (absence of coordinated atrial activity) (unpublished).

Paroxysmal atrial fibrillation (PAF) is thought to constitute between 25 and 60% of cases of AF [6] and is thought to precede sustained AF, culminating in progressive atrial electrical and structural remodelling, otherwise coined “AF begets AF.” PAF has been defined by the American Heart Association as “recurrent (two or more) episodes of AF that terminate spontaneously lasting between 30 seconds and less than seven days” [7]. An example of the initiation of PAF is seen in Figure 1.

The true prevalence of PAF is unknown due to asymptomatic episodes and a low yield afforded by conventional monitoring. However between 6 and 28% of cryptogenic strokes have been found to be secondary to PAF [8] and PAF conveys an equivalent risk of stroke to sustained forms of AF [9]. Unfortunately PAF, relative to the other forms of AF termed “persistent” and “permanent,” can be a particular challenge to diagnose due to the variable onset of the arrhythmia, potential brevity, and also frequent lack of symptoms [10].

## 2. Pathophysiology of Atrial Fibrillation

**Figure 2:**
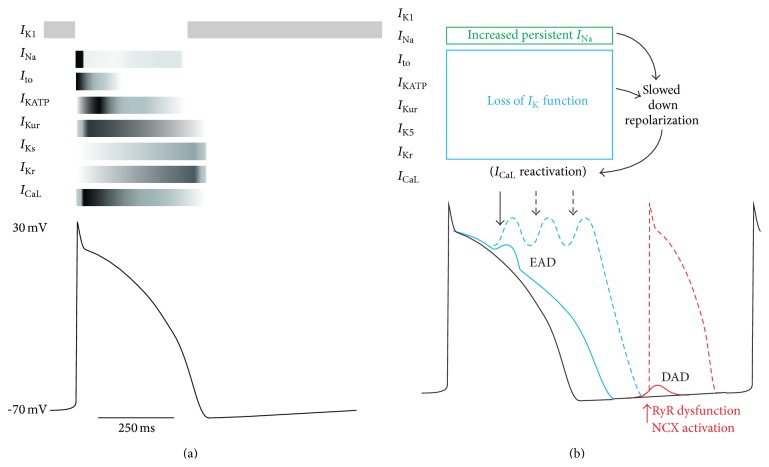
Illustrative diagram of a typical human left atrial AP with associated currents shown above (a). Electrical remodeling can result in abnormal APs (b). *I*_CaL_ reactivation (blue) can cause early-after depolarization (EAD) that may result in repetitive EAD. Alternatively spontaneous ryanodine receptor (RYR) release or Na^+^/Ca^2+^ exchanger activation (NCX) (red) would result in delayed-after depolarization (DAD) (unpublished).

The cardiac action potential (AP) is a key determinant of cardiac electrical activity and results from transmembrane ion fluxes through ion channels and transporters. A schematic representation of a human atrial action potential and the principal currents involved is shown in Figure 2(a). The resting potential is maintained at about −80 mV via K^+^ equilibrium potential. Upon activation, rapid depolarization occurs by a large inward Na^+^ current (*I*_Na_) and an inward Ca^2+^ current via L-type Ca^2+^ channels (*I*_CaL_) resulting in the plateau phase. As the Ca^2+^ current declines the AP repolarizes to the resting potential.

Arrhythmia generation in the left atrium (LA) originates from a combination of abnormalities in impulse initiation, impulse conduction, or a combination of the two [11]. Abnormal impulse initiation can be subclassified further due to enhanced automaticity or triggered activity. Enhanced automaticity (ectopic arrhythmias) may be produced by irregular intracellular Ca^2+^ release. Similarly triggered activity, caused by a spontaneous inward current, generates secondary afterdepolarizations early in the plateau phase (EADs) or upon repolarisation (DADs) (Figure 2(b)). Alternatively due to abnormal AP conduction, reentrant arrhythmias occur, preventing the normal pattern of propagation across the myocardium. This is attributable to a combination of alteration of the electrical properties of intercellular gap junctions; structural remodelling of the myocardium; and generation of EADs/DADs that* per se* have slower conduction. Both mechanisms may be present simultaneously where ectopic activity begets reentrant arrhythmias resulting in self-perpetuation.

The former described electrophysiological mechanisms are coupled to atrial electrical remodeling, characterised by changes in atrial refractoriness and slowed conduction time. These changes occur due to alterations in AP currents, especially Ca^2+^ influx and its subsequent homeostasis, and provide ongoing atrial arrhythmogenic substrates [12].

**Figure 3:**
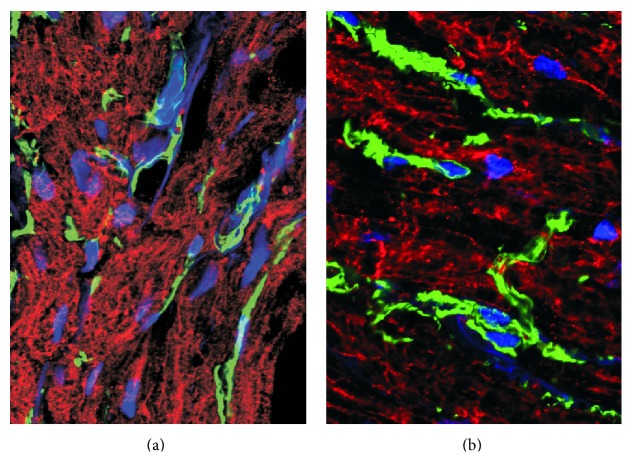
Staining of the left atrial appendage demonstrating fibrosis stained with vimentin (green) in a patient in sinus rhythm (a) and AF (b) (unpublished).

Atrial structural remodeling is typified by cardiac fibrosis, characterized by accumulation of collagenous material in the extracellular space (Figure 3), and is proposed to widen gap junctions and decrease communication between cardiomyocytes. Reduced AP conduction velocity is directly correlated to the extent of fibrosis and is linked to persistent reentrant circuit arrhythmias [13]. Angiotensin II produced by fibroblasts exacerbates the situation to increase cellular proliferation and cardiac fibrosis [14]. Alternatively structural remodeling may arise due to the release of proinflammatory cytokines after surgery or injury and the interaction of inflammatory proteins with angiotensin II promotes further atrial fibrosis [15]. This inflammatory response has been implicated in the aetiology of AF although the question remains whether this is attributable to the arrhythmia itself or another underlying disease state [16].

**Figure 4:**
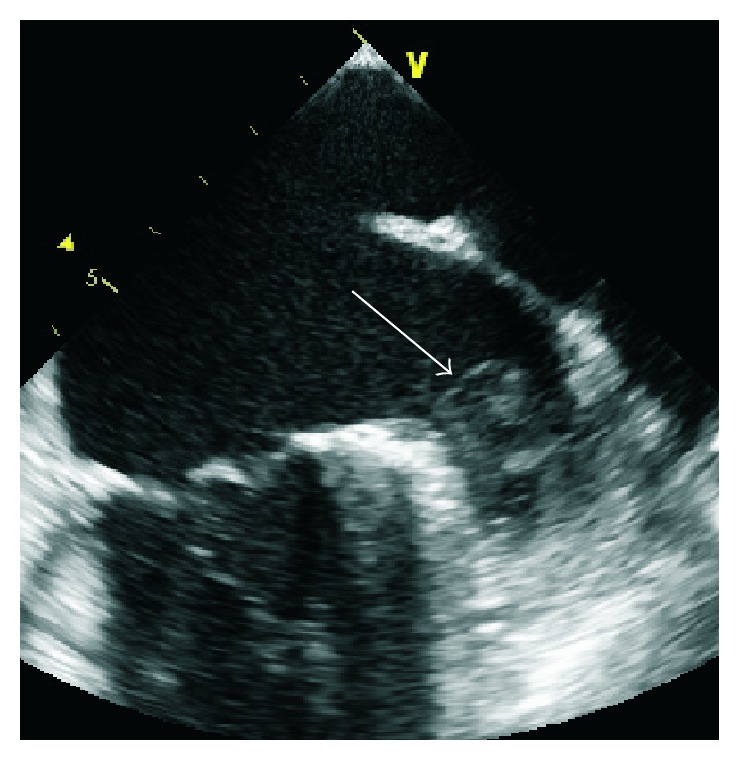
Thrombus visualized in the left atrial appendage is marked with an arrow (unpublished).

The ultimate consequences of AF, stroke and systemic embolism, result from the dislodgement of LA thrombi. Thrombus is most commonly located in the left atrial appendage (LAA) [17], a small pouch found in the LA (Figure 4). The generation of thrombi is triggered by atrial stasis, as a result of impaired atrial contraction due to atrial arrhythmia. Other factors promoting thrombus formation include damage to the atrial wall and a hypercoagulable state [18].

## 3. Current Diagnostics for Paroxysmal Atrial Fibrillation

Current methods to diagnose PAF are limited to electrocardiogram (ECG) analysis, which may fail to document an episode of AF if occurring outside the monitoring period. Recent trials have highlighted that prolonged cardiac monitoring detects significantly more cases of PAF in the cryptogenic stroke population than using a conventional 24-hour Holter monitor [19, 20]. Clinical risk factors for AF are widely recognized and models have been proposed to predict incident AF using a number of clinical variables. Such a risk score devised by the Framingham Heart Study is based on a number of simple clinical parameters including age, gender, a significant murmur, cardiac failure, systolic blood pressure, hypertension, body mass index, and PR interval. The score yielded a C-statistic of 0.78 (95% CI 0.76–0.80), with a C-statistic being a measure of a model's predictive power and a value of over 0.7 considered to be reasonable. The addition of echocardiographic measurements further improved this to 0.79 [21]. There is however a lack of diagnostic armoury to detect PAF. This is in stark contrast to other cardiovascular conditions, for example, coronary artery disease and cardiac failure, with a comparable disease prevalence and routine use of diagnostic biomarkers [22].

## 4. Biomarkers

A biomarker can be defined as “a characteristic that is objectively measured and evaluated as an indicator of normal biological processes, pathogenic processes, or pharmacologic responses to a therapeutic intervention” [23]. Biomarkers not only have the potential to identify a disease process but also provide valuable information about underlying disease mechanisms and, as a consequence, potential therapeutic targets.

Biomarkers have been proposed as a tool to predict onset of AF in a variety of settings including at initial presentation, following cardiothoracic surgery, recurrence after cardioversion and ablation, and in the event of a cryptogenic stroke. Additionally, accumulating research has shown that biomarkers could be used to predict the transition of PAF to sustained AF, as well as, alongside conventional risk scores, to determine thromboembolic risk [24, 25]. The LA has been the main focus for identifying biomarkers for PAF given the inextricable association between this chamber and the disease process itself. As such potential biomarkers to detect PAF can be broadly categorised into electrophysiological, molecular, and morphological indices.

## 5. Biomarkers for Paroxysmal Atrial Fibrillation

### 5.1. Electrophysiological Biomarkers

A number of studies have shown that simple resting ECG parameters are highly predictive of PAF. “P-maximum” is the maximum duration from the onset to the end of the P-wave deflection from all 12 ECG leads. This is seen as a marker of prolonged atrial conduction time, a hallmark of patients with AF. “P wave dispersion,” the difference between the maximum and minimum P-wave duration in any of the 12 leads, signifies nonuniform atrial conductivity. Both parameters are representative of the underlying atrial remodelling occurring in AF and previously alluded to. One study showed that P-maximum and P-wave dispersion were highly predictive of previous episodes of PAF. A P-maximum of at least 110 ms had a sensitivity of 88% and specificity of 75% and a P-wave dispersion of at least 40 ms yielded a sensitivity of 83% and specificity of 85% for PAF [26]. These findings have been substantiated by another study showing that P-wave dispersion was an independent predictor of PAF in stroke patients. In this population a P-wave dispersion 57.5 ms and above predicted PAF with a sensitivity and specificity of 80% and 73%, respectively [27].

Likewise a J-shaped relationship was shown between the QTc interval and risk of incident AF in a large primary care population. QTc intervals at the 99th percentile and above (≥464 ms) resulted in an overall hazard ratio of 1.44 for all AF subtypes. Additionally an even more powerful relationship was seen in lone AF with a QTc interval ≥458 ms demonstrating a hazard ratio of 2.32. Conversely a QTc ≤372 ms was associated with a hazard ratio of 1.45 [28]. More specific to PAF, another group have shown that a prolonged QTc interval is an independent predictor of PAF in patients presenting with ischaemic stroke. In this instance a QTc threshold ≥438 ms generated a sensitivity of 59.4% and specificity of 83.7% [29]. It has been proposed that these associations may be on account of universal expression of ion channels in both atrial and ventricular tissue.

**Figure 5:**
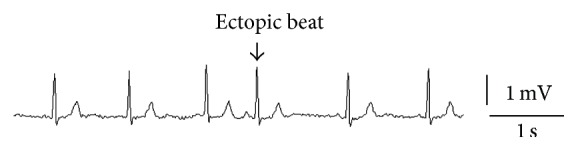
An example of an atrial premature beat is marked (unpublished).

Atrial premature beats (APBs) precede PAF in a significant proportion of AF episodes and are a feature of enhanced automaticity, a recognised mechanism for AF (Figure 5). In one study monitoring 33 patients with documented PAF and 297 total episodes of AF, the arrhythmia was initiated by APBs in 93% of cases [30]. Consequently frequent APBs have been suggested as a marker of predisposition to PAF. One study investigating 98 stroke patients with transtelephonic monitoring determined that at least 100 APBs on a 24-hour Holter monitor equated to an odds ratio of 11 for the subsequent development of PAF after one month [31].

In another cohort of stroke patients, greater than 4 APBs per hour during an initial 24-hour Holter monitor equated to subsequent development of PAF in 19.6%, compared to 2.8% in those with 4 APBs per hour or less [32]. Using a similar study design, Wallmann et al. also deduced that frequent APBs were an independent risk factor for PAF in stroke patients. Significantly more of those patients with frequent APBs (defined as at least 70 in 24 hours) developed PAF at 22.4 months compared with those with infrequent APBs (33% versus 5%) [33].

### 5.2. Molecular Biomarkers

Previously validated biomarkers, currently used in standard clinical practice to diagnose cardiac failure and myocardial infarction, have also been tested in the setting of AF. Numerous studies have shown an association between AF and brain natriuretic peptide (BNP) with persistently higher plasma levels than in healthy matched controls and a reduction to that of control subjects following successful restoration of sinus rhythm [34]. It has been proposed that the main source of BNP release in AF is the atrium as a result of pressure and volume overload. It remains to be established however whether BNP is merely a marker of atrial dysfunction or active in the underlying pathological process [35]. With a particular focus on PAF, several studies have shown a relationship between natriuretic peptides and diagnosis of this arrhythmia. One study showed higher levels of plasma BNP in patients with lone PAF compared to age- and sex-matched controls [36]. A further study confirmed significantly raised N-terminal of prohormone brain natriuretic peptide (NT-proBNP, a peptide cleaved from pro-BNP to release BNP) in contrast to matched controls. Yet a similar relationship was not demonstrated for pro-ANP (atrial natriuretic peptide) in this group [37]. Likewise in the Find-AF study, BNP levels were significantly higher in those cryptogenic stroke patients with confirmed PAF than those without [38]. A similar prospective study found that a BNP over 140 pg/mL had a sensitivity and specificity of 77.6% and 84.8%, respectively, for cardioembolic stroke [39]. Likewise NT-proBNP levels over 265.5 pg/mL conferred a sensitivity 100% and specificity 70.5% for PAF [40]. Similarly troponin, another established cardiac biomarker, is increased in PAF subjects relative to their controls. Elevation of troponin levels in cryptogenic stroke patients independently predicted new onset AF during 24-hour Holter monitoring [41]. This finding was also confirmed in a similar retrospective study [42].

Markers of inflammation, implicated in the pathogenesis of AF, have yielded mixed results. Raised serum IL-18 was significantly associated with both PAF and persistent AF with a twofold concentration in all AF subtypes compared to controls [43]. Another study confirmed that patients with PAF had significantly higher levels of CRP (C-reactive protein) than their controls, in a graded fashion according to arrhythmia burden [44]. Similarly a raised white blood cell count predisposed to incident AF after 5 years in the Framingham Study [45]. In stark contrast another study demonstrated no difference in the inflammatory markers CRP, IL-6, and IL-8 between PAF patients and their controls when attending for radiofrequency ablation [46]. Glucose haemostasis and lipid metabolism also appear to be associated with the development of AF. In the Atherosclerosis Risk in Communities (ARIC) cohort a positive linear relationship was seen between haemoglobin A1c (HbA1c) and incident AF in patients both with and without type 2 diabetes [47]. Interestingly in the Women's Health Study however, although HbA1c was positively correlated with the incidence of nonparoxysmal AF, it was inversely related to PAF [48]. Additionally the ARIC study demonstrated that both higher LDL and total cholesterol resulted in a lower incidence of all AF subtypes [49]. This, somewhat unexpected, inverse relationship was replicated by another group [50] yet an explanation for this repeated finding is not clear.

Parameters reflecting thrombogenesis, the source of cardioembolism and stroke in PAF, have also been investigated. For example, plasma von Willebrand factor and fibrinogen were significantly elevated in patients with PAF compared to their matched controls [51]. Similarly fibrinogen and fibrin D-dimer were significantly increased in PAF relative to age- and sex-matched controls [52]. Additionally regulators of extracellular turnover, the culprits of the previously described fibrotic remodelling, were all increased in lone PAF subjects compared to their controls. These markers include CICP (C-terminal propeptide of collagen type-I), CITP (C-terminal telopeptide of collagen type-I), MMP-1 (matrix metalloproteinase-1), and TIMP-1 (tissue inhibitor of matrix metalloproteinase) [53]. Likewise CITP was also raised in PAF cases compared to healthy subjects in another study [54]. Both of these studies were limited by a younger control group however, introducing potential confounding.

A genetic predisposition has been shown to contribute to the development of AF. For example, the Framingham Study showed a heightened risk of AF in the children of a parent with AF, independent of other risk factors (odds ratio of 1.85) [55]. Genetic analysis of over 14,000 European and Japanese individuals with a history of AF noted susceptibility signals on chromosome 4q25, upstream of PTIX2, a gene previously implicated in AF [56]. Furthermore six further susceptibility loci (1q24, 7q31, 14q23, 9q22, 15q24, and 10q22) were identified in a meta-analysis comprising over 10,000 individuals with AF of European decent [57]. These findings prompted the Women's Health Study to validate a prediction score for incident AF incorporating 12 single-nucleotide polymorphisms in nine loci. Genetic risk markers were shown to improve a conventional clinical risk score from a C-statistic of 0.718 to 0.741 (*P* = 0.001) [58]. Whether genetic markers will aid in the diagnosis of PAF in particular is, as yet, to be definitively answered. There is however promising research suggesting that microRNAs, stable noncoding RNAs (ribonucleic acid) found in serum and plasma which modulate RNA transcription, may play such a role. There is a relative paucity of research in this area to date and studies investigating a link to PAF are even scarcer. However promisingly, several preliminary studies have shown that plasma miRNA-150 expression, already implicated in the regulation of genes associated with atrial remodeling, is significantly reduced in individuals with PAF [59, 60].

### 5.3. Morphological Biomarkers

It is widely recognised that LA size is a powerful predictor of cardiovascular mortality, conferring an independent 2.3-fold excess risk of cardiovascular mortality at 13 years [61]. Specific to AF, each increase in LA diameter by 5 mm increased the risk of new AF by 39% in the Framingham Heart Study [62]. Likewise subjects with PAF had increased LA size relative to healthy controls with an inferosuperior dimension of at least 50 mm, yielding a sensitivity and specificity for PAF of 66% and 80%, respectively [63]. These observations are a direct result of the structural remodelling occurring in the LA in the course of the disease process.

With the advent of increasingly sophisticated techniques to assess the LA, echocardiographic indices of function are now widely thought to be more robust predictors of outcomes than size alone [64, 65]. Methods to assess LA function include Doppler analysis of transmitral flow and tissue Doppler assessment of LA myocardial velocities. Not only have alterations in LA function been shown to be a hallmark for PAF, but also they may predict the onset of AF following cardiothoracic surgery, cardioversion, and ablation, as well as being an independent marker of thromboembolic risk [66–69]. Variables of LA function, as assessed using transoesophageal echocardiography (TOE), were impaired in PAF patients following stroke. LAA peak velocity was markedly reduced in the PAF group compared to the control group. Additionally LA spontaneous echo contrast, graded from 0 (none) to 4+ (severe), was higher in the PAF group. Both of these measurements reflect atrial stasis and were more powerful predictors of PAF than LA dimension alone [70].

**Figure 6:**
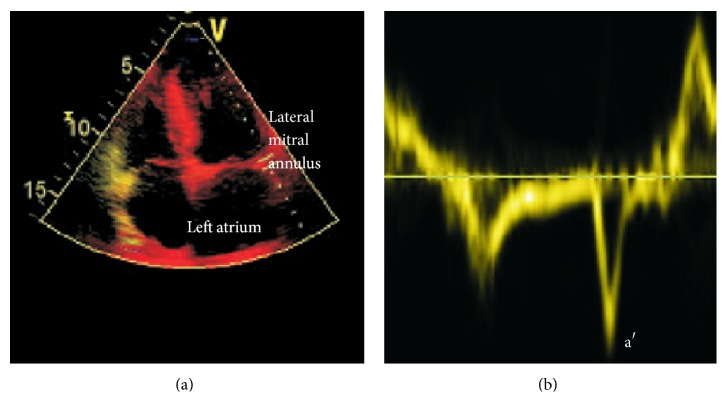
Measurement of left atrial velocity using tissue Doppler. The left image demonstrates the position of the probe at the lateral mitral annulus. The right image represents the velocities generated during diastole with the atrial component (termed a′) marked (unpublished).

These results have been replicated using transthoracic echocardiography where, LA function as assessed by myocardial Doppler, is thought to predict PAF more precisely than LA dimensions. Toh et al. used a novel marker of both LA size combined with LA pump function [LAVI/a′] to discriminate between patients with PAF in a group of hypertensive patients. LAVI (left atrial volume index) is calculated from the LA volume corrected for body surface area. Left atrial velocity in late diastole, or a′, is derived from myocardial tissue Doppler measured from the mitral annulus (Figure 6). In this population a LAVI/a′ threshold of 2.7 conferred a sensitivity 82% and specificity 91% for PAF. [71]. The Find-AF group used the same method to distinguish between stroke or TIA patients with underlying PAF. It was deduced that LAVI/a′ of 2.3 had a 93% sensitivity and 55.8% specificity. Again this parameter predicted PAF more powerfully than measures of LA size, including LA diameter and LAVI [72].

Mitral valve disease is commonly associated with a concurrent diagnosis AF, attributed to atrial remodeling and dilatation as a result of volume and pressure overload. For example, over 40% of individuals with rheumatic mitral valve disease and between 44 and 48% of those with degenerative mitral regurgitation (MR) develop AF in the long-term [73, 74]. One observational study has proposed a form of “functional MR” as a direct consequent of AF, possibly induced by atrial wall dyssynchrony, which improves following restoration of sinus rhythm [75]. As a predictive tool, one group proposed that, in patients with at least moderate MR, the rate of left ventricular pressure rise in early systole (*dP/dt* max) independently predicted new onset AF or ischaemic stroke. The study was significantly limited by a small sample size with only 9 patients having this combined end point [76]. To our knowledge no large studies assessing the use of MR as a diagnostic marker for PAF specifically have been undertaken to date.

## 6. Conclusions

Despite several decades of plausible research suggesting that biomarkers might be used to improve the diagnosis of PAF (summarised in Table 1) these techniques have not as yet entered clinic practice. This begs the question why this might be the case.

There appears to have been a shift in clinicians' and scientists' attitudes to AF in recent years and PAF is no longer seen as a benign entity. This and the impending “AF epidemic” have highlighted the importance of optimal arrhythmia detection. This had been somewhat neglected previously with the 24-hour Holter monitor remaining commonplace as a first-line investigation. Critically the lack of a universal definition for PAF has hindered research in the field. AF research is riddled with conflicting definitions primarily dependent on the duration of the arrhythmia and tendency to self-terminate. Furthermore a lack of consensus may be due to oversimplified classification systems. AF is a heterogeneous condition likely to represent a number of underlying pathologies and a lack of aetiological classification may have hindered the quest for improved diagnostic markers.

The majority of the literature in this review suggests that biomarkers might be a valuable addition to current investigational techniques. However these findings are admittedly subject to inherent publication bias. Furthermore recurrent methodological flaws are encountered. Many of the papers published in the area consist of small populations. Additionally a number of groups relied on self-reported AF as opposed to objective screening. Crucially many of the control groups were younger than those with confirmed PAF. Given that age is an independent risk factor for AF and with age comes increasing comorbidity, this introduces confounding. Lastly a number of the techniques described are time-consuming and some echocardiographic techniques are hindered by difficult patient anatomy.

In summary it remains an open question if biomarkers will add to conventional diagnostic techniques for PAF. If the role of biomarkers is to be established then future research requires refinement. We propose that an accurate future biomarker is likely to feature a combination of electrophysiological, molecular, and morphological indices therefore encompassing the heterogeneity seen in this condition. A reliable diagnostic marker to improve the diagnosis of undetected PAF and ultimately reduce risk of consequent thromboembolism certainly warrants further investigation.

## Figures and Tables

**Table 1:** A summary of the main advantages and disadvantages of current diagnostic techniques for PAF (not bold) including potential biomarkers for PAF based on the evidence detailed in this review (bold) (unpublished).

Diagnostic class	Technique	Sensitivity	Specificity	Automated analysis	Remote analysis	Cost	Advantages	Disadvantages
ECG rhythm monitoring	Continuous long-term ECG monitoring using implantable devices	High	High	+	+	High	High sensitivity and specificity	Costly, invasive equipment required
	Medium-term noninvasive ECG monitoring >24 hours	Moderate	High	+/−	+/−	Moderate	Moderate sensitivity and specificity	Patient inconvenience, some cases missed
	Short-term ECG monitoring -24 hours	Low	High	+/−	+/−	Low to moderate	Relatively inexpensive	Low diagnostic yield

Electrophysiological Biomarkers	**Analysis of ECG indices**, **for example, P wave dispersion, QTc interval**	**Low to high**	**Moderate to high**	**−**	**−**	**Low**	**Cost-effective test already in routine clinical practice**	**Relatively labour intensive without automated analysis. Room for potential error**
	**Frequency of atrial premature beats on 24-hour** **Holter monitor**	**Moderate**	**Low-moderate**	**+/−**	**+/−**	**Low to moderate**	**Noninvasive test which the patient may already be undergoing**	**Low specificity**

Molecular biomarkers	**Proteins**	**Moderate to high**	**Moderate**	**+**	**−**	**Low**	**Mass screening possible**	**Relatively low specificity**
	**microRNAs**	**Expected to be equivalent to protein biomarkers**						

Morphological biomarkers	**Echocardiography LA size **	**Low**	**Moderate**	**−**	**−**	**Moderate**	**Noninvasive test which the patient may already be undergoing. LA size is a standard measurement**	**Highly trained specialists and costly equipment required**
	**Echocardiography LA function**, **for example, myocardial tissue Doppler**	**High**	**Moderate**	**−**	**−**	**Moderate**	**Noninvasive test which the patient may already be undergoing**	**Highly trained specialists and costly equipment required. Potential room for error in patients with suboptimal image quality**
